# Description and Nomenclature of *Neisseria meningitidis* Capsule Locus

**DOI:** 10.3201/eid1904.111799

**Published:** 2013-04

**Authors:** Odile B. Harrison, Heike Claus, Ying Jiang, Julia S. Bennett, Holly B. Bratcher, Keith A. Jolley, Craig Corton, Rory Care, Jan T. Poolman, Wendell D. Zollinger, Carl E. Frasch, David S. Stephens, Ian Feavers, Matthias Frosch, Julian Parkhill, Ulrich Vogel, Michael A. Quail, Stephen D. Bentley, Martin C.J. Maiden

**Affiliations:** University of Oxford, Oxford, UK (O.B. Harrison, J.S. Bennett, H.B. Bratcher, K.A. Jolley, M.C.J. Maiden);; University of Würzburg, Würzburg, Germany (H. Claus, M. Frosch, U. Vogel);; The Sanger Institute, Cambridge, UK (Y. Jiang, C. Corton, J. Parkhill, M.A. Quail, S.D. Bentley);; National Institute for Biological Standards and Control, Potters Bar, UK (R. Care, I. Feavers);; Crucell, Leiden, the Netherlands (J.T. Poolman);; Walter Reed Army Institute of Research, Silver Spring, Maryland, USA (W.D. Zollinger);; Frasch Biologics Consulting, Martinsburg, West Virginia, USA (C.E. Frasch);; Emory University, Atlanta, Georgia, USA (D.S. Stephens)

**Keywords:** Neisseria meningitidis, capsule, serogroup, bacteria, nomenclature

## Abstract

Pathogenic *Neisseria meningitidis* isolates contain a polysaccharide capsule that is the main virulence determinant for this bacterium. Thirteen capsular polysaccharides have been described, and nuclear magnetic resonance spectroscopy has enabled determination of the structure of capsular polysaccharides responsible for serogroup specificity. Molecular mechanisms involved in *N. meningitidis* capsule biosynthesis have also been identified, and genes involved in this process and in cell surface translocation are clustered at a single chromosomal locus termed *cps*. The use of multiple names for some of the genes involved in capsule synthesis, combined with the need for rapid diagnosis of serogroups commonly associated with invasive meningococcal disease, prompted a requirement for a consistent approach to the nomenclature of capsule genes. In this report, a comprehensive description of all *N. meningitidis* serogroups is provided, along with a proposed nomenclature, which was presented at the 2012 XVIIIth International Pathogenic Neisseria Conference.

Thirteen *Neisseria meningitidis* serogroups have been described on the basis of serologic differences of the capsule; of these 13 serogroups, 6 (A, B, C, W, X, Y) cause invasive meningococcal disease. The polysaccharide capsule is a key virulence determinant, and for serogroups A, C, W, and Y, it forms the basis of polysaccharide conjugate vaccines. In one of the first reports distinguishing *N. meningitidis*, disease isolates were serologically classified into types I–IV on the basis of agglutination reactions with immune rabbit serum (*1*). In 1950, the subcommittee on *Neisseria* of the Nomenclature Committee of the International Association of Microbiologists recommended that types I and III be combined into serogroup A; type II become serogroup B; a type II subgroup, termed type II-α, become serogroup C; and type IV become serogroup D. After the report of a fourth serogroup, Z′ (later shown to be serogroup E), 3 new serogroups (X– Z) were identified by using double agar diffusion (*2*,*3*). In 1981, three more serogroups (H, I, K) were proposed, and a fourth (serogroup L) was identified in 1983 (*4*,*5*).

Nuclear magnetic resonance spectroscopy enabled determination of the structure of capsular polysaccharides responsible for serogroup specificity, and structures for 12 of the 13 serogroups (all but serogroup D) from *N. meningitidis* capsular polysaccharides have been reported (*6*–*15*). Molecular mechanisms of capsular polysaccharide synthesis have been elucidated; genes involved in polysaccharide biosynthesis and cell surface translocation are clustered at a single chromosomal locus termed *cps.* Genes within this locus are divided into 6 regions: A–D, D′, and E (*16*). Genes in region A encode enzymes for biosynthesis of the capsular polysaccharide, and genes in regions B and C are implicated in the translocation of the high molecular weight polysaccharides to the cell surface.

Complete nucleotide sequences of *cps* loci encoding serogroups A–C, W, and Y have been elucidated. Serogroup-specific capsule biosynthesis genes located in region A have been published for serogroup X, and nucleotide sequences for serogroups E, L, and Z have been submitted to GenBank (accession nos. AJ576117, AF112478, and AJ744766, respectively) (*17*–*19*). This study provides a comprehensive description of all *N. meningitidis* serogroups and presents proposed revisions to the nomenclature.

## Materials and Methods

### Strain Selection

Serogroup D, H, I, and K isolates were obtained from a collection maintained at the National Institute for Biological Standards and Controls, Potters Bar, UK; the isolates were originally from a collection (1980s) from the People’s Republic of China Committee for Culture Collection of Microorganisms. Serogroup E, W, X, and Y isolates were from the Bavarian *N. meningitidis* carriage collection (*20*). Three serogroup L isolates were analyzed with additional serogroup H, I, and K isolates obtained from Paula Kriz (Czech Republic), who had obtained the isolates from Fraser Ashton, who had acquired them from People’s Republic of China (Table 1). The sequenced genomes from serogroup A isolate Z2491, serogroup B isolate H44/76, and serogroup C isolates FAM18 and 053442 were used (Table 1) (*21*–*24*). Isolates were grown on Mueller-Hinton agar supplemented with 5% (vol/vol) defibrinated sterile horse blood for 15 h at 37°C in a 5% (vol/vol) CO_2_ atmosphere. DNA was extracted by using the Wizard Genomic DNA Purification Kit (Promega, Southampton, UK) according to the manufacturer’s instructions.

**Table 1:** Description of *Neisseria meningitidis* isolates*

Isolate name	Serogroup	Origin	Disease status	Strain designation	GenBank accession no.
*N. meningitidis* Z2491	A	The Gambia	Invasive	A:P1.7, 13–1: F1–5: ST-4 (cc4)	AL157959
*N. meningitidis* H44/76	B	Norway	Invasive	B:P1.7,16: F3–3: ST-32 (cc32)	CP002420
*N. meningitidis* FAM18	C	United States	Invasive	C:P1.5,2: F1–30: ST-11 (cc11)	AM421808
*N. meningitidis* 053442	C	PRC	Invasive	C:P1.7–2,14: F3–3: ST-4821 (cc4821)	CP000381
*N. meningitidis* 29013	D	PRC	Unknown	C:P1.22,14–6: F3–16: ST-8723 (cc213)	ERR028660
*N. meningitidis* α707	E	Germany	Carrier	E:P1.5–1,2–2: F4–3: ST-254 (cc254)	HF562982
*N. meningitidis* 29031	H	PRC	Carrier	H:P1.21,3: F4–21: ST-4959 (-)	ERR028662
*N. meningitidis* H-ASH/87	H	PRC	Carrier	H:P1.21,3: F4–21: ST-4959 (-)	ERR036095
*N. meningitidis* H-ZANE/83	H	PRC	Carrier	H:P1.21,3: F4–21: ST-4959 (-)	ERR036096
*N. meningitidis* 29043	I	PRC	Carrier	I:P1.22,14–6: F5–2: ST-5594 (-)	ERR028663
*N. meningitidis* I-ZANE/87	I	PRC	Carrier	I:P1.22,14–6: F5–2: ST-5594 (-)	ERR036097
*N. meningitidis* 29046	K	PRC	Carrier	K:P1.7–2,14: F5–14: ST-8724 (-)	ERR028664
*N. meningitidis* K-ASH/87	K	PRC	Carrier	K:P1.7–2,14: F5–14: ST-8724 (-)	ERR036087
*N. meningitidis* WUE3608	L	Unknown	Carrier	L:P1.18–1,3: F1–5: ST-963	HF562986
*N. meningitidis* L-ASHTON/87	L	PRC	Carrier	L:P1.22,14: F1–38: ST-8902 (-)	ERR036088
*N. meningitidis* 21033	L	Dublin, Ireland, UK	Carrier	L:P1.7–2,13–1:F1–5: ST-3258 (-)	ERR063490
*N. meningitidis* α275	W	Germany	Carrier	W:P1.18–1,3: F4–1: ST-22 (cc22)	HF562987
*N. meningitidis* WUE171	W	Germany	Unknown	W:P1.5–9, 10: F3–6: ST-11 (cc11)	HF562992
*N. meningitidis* α388	X	Germany	Carrier	X:P1.5–1,2–2: F5–1: ST-765 (cc254)	HF562988
*N. meningitidis* α162	Y	Germany	Carrier	Y:P1.5–2,10–1: F4–1: ST-23 (cc23)	HF562989
*N. meningitidis* WUE172	Y	Unknown	Unknown	Y:P1.5,2: F1–1: ST-166 (cc11)	HF562992
*N. meningitidis* WUE173	Z	Unknown	Unknown	Z:P1.7–1,1: F1–7: ST-4443 (-)	HF562991

*PRC, People’s Republic of China; UK, United Kingdom.

### PCR and Nucleotide Sequencing of the Capsule Locus

The genes for the *N. meningitidis cps* locus are located between a gene encoding a putative inner membrane transport protein (NMC0044 FAM18 genome annotation) and a gene encoding the sodium/glutamate symport carrier protein, *gltS* (NMC0069). PCR reactions were performed for isolates 29013, α707, 29031, 29043, 29046, 3608, α275, α388, α162, WUE171, WUE172, and WUE173 (Table 1) by using the Expand Long Template PCR System (Roche Applied Science, Burgess Hill, UK) according to the manufacturer’s recommended protocol, with annealing at 55°C and extension at 68°C for 20 min. Initial reactions used 1 of the following primer pairs: *gltS* (NMC0069) to *tex* (NMC0059) (primers *gltS* 5′- CCGACCAAGCCGTATTGC + ATGATACTCGAAGGCGTGGTT-3′ and *tex* 5′- TGTCGAAGCCGTCCATAATCT + GCCCTGTCCAACAAGTTCGT-3′) and *tex* to NMC0044 (primers *tex* 5′- CGCCCGGTTCGTCATCC + TTGCTGCTGGTAGGCGAATCC -3′ and NMC0044 5′- CGGGCGAACACGGTAAT + TATCGTTGGTGCGCTGGTTAT-3′).

### Genome Sequencing

Genomic data for serogroups D, H, I, K, and L were obtained by using the Illumina sequencing platform and de novo assembly (Illumina, San Diego, CA, USA), using the shuffle and Velvet Optimization scripts found within Velvet 1.1.03 (*25*) (Table 1). By using VelvetOptimiser version 2.1.7 (http://bioinformatics.net.au/software.velvetoptimiser.shtml), optimal k-mer lengths ranging from 41 to 65 were selected and contigs were generated. These contigs were deposited in the Bacterial Isolate Genome Sequence Database (BIGS_DB_) along with the isolate provenance and name, after which contigs were scanned for genes within the *cps* locus and tagged and alleles were assigned (*26*). New alleles were manually checked for a correct start and stop codon and aligned with known alleles before assignation. Additional data (e.g., multilocus sequence type [MLST]), and PorA, PorB, and FetA designations were obtained.

### Annotation and Bioinformatic Methods

Predicted proteins were clustered into homology groups by using TribeMCL algorithm with a cutoff of 1e-70 (http://doc.bioperl.org/bioperl-run/lib/Bio/Tools/Run/TribeMCL.html#General). The genes within the *cps* loci that encoded proteins with the same homology group were assigned the same name, exceptions being those genes found in Region A. BLASTp (http://blast.ncbi.nlm.nih.gov/Blast.cgi?PAGE=Proteins) searches were done by using a reference sequence database with default settings.

Annotation was performed by using the genome viewer Artemis (*27*). The *cps* nucleotide sequence locus for each serogroup was stored in BIGS_DB_ and linked with the corresponding isolate record.

MEGA5 (http://megasoftware.net/) was used to calculate overall *p-*distances and G+C content and to obtain the number of polymorphisms observed within each gene. The *cps* loci were compared by using the Artemis Comparison Tool (www.sanger.ac.uk/resources/software/act/).

## Results and Discussion

### General Organization

All of the serogroups examined had comparable *cps* loci, and regions occurred in the following order D-A-C-E-D′-B, revealing a conserved gene synteny and a replication of the genomic rearrangements (Figure). Genes in regions B–D, D′, and E were conserved, whereas genes in region A were diverse; this finding is consistent with the distinct biochemical composition found within each serogroup (Table 2).

**Figure F1:**
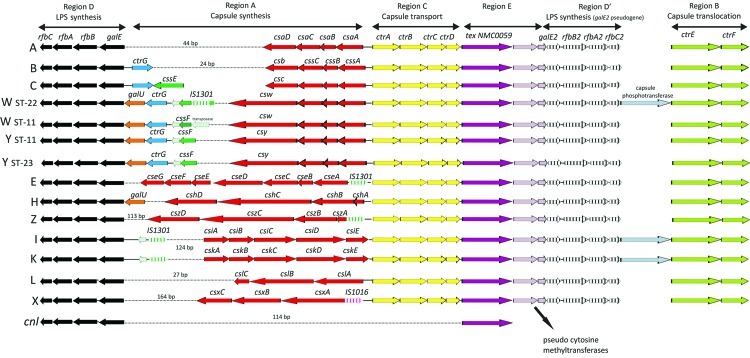
Genetic organization of the *cps* locus among *Neisseria meningitidis* serogroups A (*N. meningitidis* Z2491); B (*N. meningitidis* H44/76); C (*N. meningitidis* FAM18, 053442, and 29013); W α275 (clonal complex sequence type [ST] 22); W WUE171 (clonal complex ST-11); Y α162 (clonal complex ST-11); Y WUE172 (ST-23); E (*N. meningitidis* α707); H (*N. meningitidis* 29031); I (*N. meningitidis* 29043); K (*N. meningitidis* 29046); L (*N. meningitidis* WUE3608); X (*N. meningitidis* α388); and Z (*N. meningitidis* WUE173); and a *cnl N. meningitidis* isolate. Letters on left represent serogroups. Arrows depict gene orientation.

**Table 2:** Genetic diversity of *Neisseria meningitidis* *cps* genes*

Region, locus	Size, bp	No. alleles	No. polymorphisms (%)	*p-*distance	*d*N/*d*S ratio	G+C content
MLST						
* abcZ*	433	11	52 (11)	0.046	0.04	51
* adk*	465	7	18 (4)	0.015	0.02	52
* aroE*	490	11	131 (7)	0.102	0.28	56
* fumC*	465	12	27 (6)	0.02	0.02	57
* gdh*	501	8	18 (4)	0.014	0.05	52
* pdhC*	480	13	61 (13)	0.041	0.08	56
* pgm*	450	10	62 (14)	0.055	0.11	54
Region B						
* ctrE*	2,115	13	386 (18)	0.061	0.2	51
* ctrF*	1,260	12	59 (5)	0.012	0.14	49
Region C						
* ctrA*	1,179	11	175 (15)	0.032	0.18	47
* ctrB*	1,164	8	264 (23)	0.051	0.10	46
* ctrC*	726	8	177 (24)	0.06	0.12	43
* ctrD*	651	7	87 (13)	0.057	0.1	46
Region D						
* rfbA*	867	13	75 (9)	0.034	0.26	53
* rfbB*	1,083	15	312 (28)	0.067	0.12	53
* rfbC*	558	14	124 (220	0.09	0.22	55
* galE*	1,020	15	382 (37)	0.119	0.12	50
Region E						
* tex*	2,274	13	173 (8)	0.019	0.09	59
NMC0060	1,007	8	24 (2.4)	0.003	0.91	40
NMC0061	345	5	24 (7)	0.013	0.19	36

*Genes in region A were too diverse among serogroups for direct comparison. Region D′ was omitted because *galE2* is truncated and *rfbA*, *rfbB*, and *rfbC* were duplicates. MLST, multilocus sequence type.

The lowest GC content was in regions A and C, indicative of genes in these regions resulting from horizontal recombination (Table 2; Table 3, Appendix); regions B, D, and E displayed GC contents of 50%, 52%, and 52%, respectively, similar to those found in the *Neisseria* genomes (*21*,*22*,*33*). Bacterial genomic GC content varies considerably among species but remains uniform within a bacterial genome, such that genes acquired through horizontal genetic exchange will have a GC content different from the overall GC content found within the genome.

**Table 3:** Nomenclature of *Neisseria meningitidis* capsule genes and description*

Region, serogroup	Proposed nomenclature (NEIS)†	Previous nomenclature	Protein function	Size, bp	G+C content, %	Ref
Region A						
A	*csaA* (NEIS2157)	*mynA/sacA*	UDP-*N*-acetyl-D-glucosamine 2-epimerase	1,119	25	(*18*)
	*csaB* (NEIS2158)	*mynB/sacB*	Polymerase linking *N-*acetyl-D-mannosamine-1-phosphate monomers	1,638	28	(*18*)
	*csaC* (NEIS2159)	*mynC/sacC*	*O-*acetyltransferase	744	30	(*28*)
	*csaD* (NEIS2160)	*mynD/sacD*	Capsule transport	864	35	(*18*)
B, C, W, Y	*cssA* (NEIS0054)	*siaA/synX/synA/neuC*	*N-*acetylglucosamine-6-P 2-epimerase	1,134	31	(*17*)
	*cssB* (NEIS0053)	*siaB/synB/neuA*	CMP*-N-*acetylneuraminic acid synthase	687	41	
	*cssC* (NEIS0052)	*siaC/synC/neuB*	*N-*acetylneuraminic acid synthetase	1,050	40	
	*csb* (NEIS2161)	*siaD_B_/synD*	Polysialyltransferase	1,488	28	
	*csc* (NEIS0051)	*siaD_C_*/*synE*	Polysialyltransferase	1,479	31	
	*csy* (NEIS2163)	*siaD_Y_/synF*	Polymerase linking glucose and *N-*acetylneuraminic acid	3,114	31	
	*csw* (NEIS2162)	*siaD_W_/synG*	Polymerase linking galactose and *N-*acetylneuraminic acid	3,114	31	
	*cssE* (NEIS0050)	*oatC*	*O-*acetyltransferase	1,383	29	(*29*)
	*cssF* (NEIS2164)	*oatWY*	*O-*acetyltransferase	636	33	(*29*)
	*ctrG* (NEIS0049)	*ctrG/NMB0065*	Putative role in surface expression of sialic capsules	957	33	(*30*)
E	*cseA* (NEIS2165)	*cap29eA*	Unknown	1,197	33	
	*cseB* (NEIS2166)	*cap29eB*	Unknown	639	38	
	*cseC* (NEIS2167)	*cap29eC*	Glycosyltransferase	1,461	40	
	*cseD* (NEIS2168)	Fusion of *cap29eD* and *cap29eE*	Glycosyltransferase	2,198	39	
	*cseE* (NEIS2169)	*cap29eF*	3-deoxy-D-manno-octulosonic acid 8-phosphate synthase	852	28	
	*cseF* (NEIS2170)	*cap29eG*	CMP-2-keto-3-deoxyoctulosonic acid synthetase and 3-deoxy-D-manno-octulosonate 8-phosphatase	1,290	26	
	*cseG* (NEIS2171)	*cap29eH*	D-arabinose 5-phosphate isomerase	947	28	
H	*cshA* (NEIS2173)	–	Glycerol-3-phosphate cytidylyltransferase	399	29	
	*cshB* (NEIS2174)	–	Putative phosphotransferase with licD domain (involved in phosphorylcholine decoration of teichoic acid)	1,269	33	
	*cshC* (NEIS2177)	–	Teichoic acid synthase	3,453	34	
	*cshD* (NEIS2178)	–	Unknown, no putative conserved domains	1,035	38	
I	*csiA* (NEIS2179)	–	UDP-N-acetylglucosamine 2-epimerase	1,125	36	
	*csiB* (NEIS2180)	–	UDP-*N*-acetyl-D-mannosamine dehydrogenase	1,269	45	
	*csiC* (NEIS2181)	–	Glycosyltransferase group 1	2,289	36	
	*csiD* (NEIS2182)	–	Glycosyltransferase group 2	2,520	34	
	*csiE* (NEIS2183)	–	Putative glycosyl transferase group 1	972	42	
K	*cskA* (NEIS2179)	–	UDP-*N*-acetylglucosamine 2-epimerase	1,125	36	
	*cskB* (NEIS2180)	–	UDP-*N*-acetyl-D-mannosamine dehydrogenase	1,269	45	
	*cskC* (NEIS2190)	–	Glycosyltransferase group 1	2,289	36	
	*cskD* (NEIS2191)	–	Glycosyltransferase group 2	2,520	34	
	*cskE* (NEIS2183)	–	Putative glycosyl transferase group 1	972	42	
L	*cslA* (NEIS2184)	*lcbA*	Capsule phosphotransferase	1,101	30	
	*cslB* (NEIS2185)	*lcbB*	Capsule polymerase	2,634	28	
	*cslC* (NEIS2186)	*lcbC*	Acetyltransferase	651	39	
X	*csxA* (NEIS2187)	*xcbA*	Capsule polymerase	1,461	39	(*19*)
	*csxB* (NEIS2188)	*xcbB*	Unknown	1,053	39	
	*csxC* (NEIS2189)	*xcbC*	Unknown	771	35	
Z	*cszA* (NEIS2173)	*capZA*	Glycerol-3-phosphate cytidylyltransferase	399	43	
	*cszB* (NEIS2174)	*capZB*	Putative phosphotransferase with licD domain (involved in phosphorylcholine decoration of teichoic acid)	1,290	45	
	*cszC* (NEIS2175)	*capZC*	Teichoic acid synthase	3,825	36	
	*cszD* (NEIS2176)	*capZD*	Unknown, no conserved domains detected	1,626	38	
Region B	*ctrE* (NEIS0066)	*lipA/ctrE*	Capsule translocation	2,115	51	(*31*)
	*ctrF* (NEIS0067)	*lipB/ctrF*	Capsule translocation	1,260	49	(*31*)
Region C	*ctrA* (NEIS0055)	*ctrA*	Capsule polysaccharide export outer membrane protein	1,179	47	(*32*)
	*ctrB* (NEIS0056)	*ctrB*	Capsule polysaccharide export inner membrane protein	1,164	46	
	*ctrC* (NEIS0057)	*ctrC*	Capsule polysaccharide export inner membrane protein	726	43	
	*ctrD* (NEIS0058)	*ctrD*	Capsule polysaccharide export ATP binding protein	651	46	

*Ref, reference; –, no previous nomenclature. Blank cells in last column indicate no reference. †In addition to the common gene name, these loci are allocated a value-free nomenclature consistent with the FAM18 genome annotation but using the prefix NEIS instead of NMC.

### Nomenclature

Reports identifying genes and proteins involved in *N. meningitidis* capsule biosynthesis, combined with the increasing availability of bacterial genomes, have necessitated a more unified approach to the nomenclature of genes within *N. meningitidis* *cps* locus (Table 3, Appendix). The capsule locus from each serogroup has been uploaded to the BIGS_DB_ platform, enabling sequences from each gene within the *N. meningitidis cps* locus to be indexed and multiple *cps* loci to be typed. However, during the process, it was found that the nomenclature of some genes within this locus posed a problem. For instance, genes within region B were thought to encode lipidation enzymes and, thus, have been known as *lipA* and *lipB*. However, within the annotated genomes from *N. meningitidis* FAM18, MC58, Z2491, and 05342 and from *N. lactamica* 020–06, two other distinct *lipA* and *lipB* genes have been described encoding a lipoic acid synthetase and a lipoate-protein ligase protein, respectively. Furthermore, there are, in some instances, multiple names for the same gene.

Continued surveillance of meningococcal disease combined with the use of multiple names for some genes made it apparent that a consistent approach to the nomenclature of capsule genes was needed. To meet that need, we propose a comprehensive description of all *N. meningitidis* serogroups and a nomenclature for the *cps* locus, which was presented at the 2012 XVIIIth International Pathogenic Neisseria Conference (Table 3, Appendix).

We propose that the capsule biosynthesis genes within region A should be termed cs (for capsule synthesis) followed by a letter representing the serogroup and by a capital letter defining each gene according to the Demerec system of genetic nomenclature (*34*). For example, serogroup A capsule biosynthesis genes would be termed *csaA*–*D* (*cs* for capsule synthesis and *a* for serogroup A) (Table 3, Appendix). Under this proposal, the sialic acid capsule biosynthesis genes would be termed *cssA*–*C* (*cs* for capsule synthesis and *s* for sialic acid capsule).

Serogroups B, C, W, and Y are commonly associated with invasive meningococcal disease, and rapid diagnosis of the serogroup is key in monitoring the epidemiology of the disease and in developing prevention strategies. Thus, it is necessary to have nomenclature that identifies the serogroup simply and quickly. The polysialyltransferase genes belonging to serogroups B and C share >70% sequence identity and should be termed *csb* and *csc*, respectively. The equivalent gene in serogroups W and Y is also a sialyltransferase, but it also has a glycosyltransferase function and is distinct from the serogroups B and C genes. These distinctions should be reflected in the nomenclature: we propose that the gene for serogroups W and Y be termed *csw* and *csy*, respectively. The *O*-acetyltransferases should be termed *cssE* for serogroup C and *cssF* for serogroups W and Y; the nomenclature for *ctrG*, which has been shown to have a role in surface translocation of sialic capsules, should be retained (*30*). The remaining serogroups would also follow this scheme (Table 3, Appendix). Genes within region C that encode the capsule transport genes have been termed *ctrA*–*D* (after capsule transport), and this nomenclature should be preserved (Table 3, Appendix) (*32*). Genes within region B that are involved in capsule translocation should be designated *ctrE* and *ctrF* (*31*). Capsule genes are accessible through the PubMLST database (http://pubmlst.org/neisseria). In addition to the common gene name, these loci are allocated a value-free nomenclature consistent with the FAM18 genome annotation but using the prefix NEIS instead of NMC (Table 3, Appendix).

The W-135 and 29E serogroup designations originated at the Walter Reed Army Institute of Research as a result of a paper published by Evans et al. (*35*). We propose to rename these W and E because the numbers are historic and supply no useful information.

### Region A Capsule Biosynthesis Genes

#### Serogroup A

The serogroup A capsule is composed of repeating units of *O*-acetylated (α1→6)-linked *N*-acetyl-D-mannosamine-1-phosphate (*13*). The capsule biosynthesis region is 4,365 bp long and contains 4 genes: *csaA*–*D (*also known as *mynA*–*D* or *sacA*–*D*) (*18*,*28*). The first gene, *csaA,* encodes the UDP-*N*-acetyl-D-glucosamine (UDP-GlcNAc) 2-epimerase, which converts UDP-GlcNAc into UDP-*N*-acetyl-D-mannosamine (UDP-ManNAc); *csaB* is the polymerase linking ManNAc-phosphate monomers together with *csaC*, encoding an *O*-acetyltransferase, which transfers acetyl groups to ManNAc (*18*,*28*). The fourth gene, *csaD*, is predicted to be involved in either capsule transport or in cross-linking of the capsule to the meningococcal cell surface. The serogroup A *cps* did not contain insertion sequences, and genes within region A contained some of the lowest GC content values among all serogroups (Table 3, Appendix).

#### *Serogroups B, C, W,* and *Y*

Capsular polysaccharides from serogroups B, C, W, and Y are composed of sialic acid derivatives; serogroups B and C express (α2→8)- and (α2→9)-linked sialic acid homopolymers, and alternating sequences of D-galactose or D-glucose and sialic acid are expressed by serogroups W and Y (*6*,*7*). Region A is 5,313 bp long in serogroup B, 6,690 bp long in serogroup C, and averages ≈7,581 bp in serogroups W and Y.

All 4 serogroups contain the conserved *cssA–C* genes for cytidine-5′-monophosphate-*N*-acetylneuraminic acid synthesis; other designations for *cssA–C* include *siaA*–*C*, *synA*–*C,* and *neuA*–*C* (*17*). These are followed by *csb*, *csc*, *csw*, and *csy* genes (*siaD,* *siaDBCWY, or synD*–*G*), which are the capsule polymerases and determine the functional and nucleotide specificity for the 4 serogroups.

Serogroups C, W, and Y contain *O*-acetyltransferase genes termed *cssE* (*oatC*) and *cssF* (*oatWY*) (*29*). The 2 sequenced serogroup C and serogroup Y isolates harbored functional *cssE/cssF* genes with an *IS1301* transposase adjacent to *cssF* in serogroup Y. Serogroup W *cssF* was interrupted by the insertion sequence *IS1301*. Another gene, *ctrG*, found in all 4 serogroups, encoded a protein essential in enabling the correct expression of sialic acid polysaccharides (*30*). The orientation of this gene differed between serogroups such that it was in the same direction as the *css* genes in serogroups B and C and the opposite in serogroups W and Y (Figure).

Serogroups W and Y contained an additional gene in region A, *galU*, encoding UTP-glucose-1-phosphate uridylyltransferase, which was also found in region A of serogroup H. BLASTp searches of the derived amino acid sequence of GalU from serogroups W, Y, and H revealed that this protein shared an average of 49% sequence identity with GalU proteins from *Streptococcus pneumoniae* isolates. GalU has an essential function in capsule formation among *S. pneumoniae* isolates, catalyzing the reversible formation of UDP-Glc (uridine diphosphate glucose) and inorganic pyrophosphate from UTP (uridine 3-phosphate) and glucose 1-phosphate, and a role in virulence has been recognized for GalU in several bacterial species (*36*). Additional *galU* genes were found adjacent to the gene *argH* encoding argininosuccinate lyase in the sequenced genomes from isolates α275, 29031, H-ASH/87, and H-ZANE/83, and although the genes were conserved, they were not identical to the *galU* genes found within the *cps* locus. The genomes belonging to *N. meningitidis* Z2491, MC58, FAM18, and 053442; *N. lactamica* 020–06; and *N. gonorrhoeae* FA1090 also contained *galU* genes adjacent to *argH*. These were highly conserved (*p-*distance = 0.015) but were more distantly related to those found within the *cps* locus (*p-*distance = 0.180), indicating a different origin for *cps*-associated *galU* genes.

The serogroup D isolate was found to contain serogroup C capsule biosynthesis genes (Figure and Technical Appendix Figure 1, panel A). The *cps* locus from the prototype serogroup D isolates deposited by Gordon and Murray in 1917 and Branham in 1928 also contained serogroup C-specific capsule genes; however, neither isolate gave precipitins with antiserum to serogroup C, suggesting that these isolates were unencapsulated (C. Frasch. pers. comm.). The presence of internal stop codons in the *ctrA* and *ctrE* genes is consistent with this and further confirms that serogroup D does not exist.

#### Serogroup E

The serogroup E capsule consists of alternating D-galactosamine and 2-keto-3-deoxyoctulosonate (KDO) residues (*9*). Region A is ≈9,613 bp long, including *IS1301*, and contains 7 genes, *cseA–G* (formerly *29eA*–*H*); gene *cseE* encodes a putative 3-deoxy-phosphooctulonate synthase, *cseF* encodes a putative 3-deoxy-D-manno-octulosonate cytidylyltransferase, and *cseG* encodes a D-arabinose 5-phosphate isomerase protein. The deduced amino acid sequence from *cseE* shared 83% sequence identity with a 3-deoxy-8-phosphooctulonate synthase belonging to *N. elongata* subsp. *glycolytica* and 81% identity with *N. subflava* NJ9703; *cseF* shared 66% sequence identity with a putative 3-deoxy-D-manno-octulosonate cytidylyltransferase belonging to multiple bacterial species, including *Pseudomonas putida* and *Escherichia coli*. The genes *cseD* and *cseE* were found to be fused and were predicted to form 1 protein; this prediction is in agreement with the suggestion that the genes for KDO synthesis were dispensable probably because of complementation of lipopolysaccharide–KDO synthesis elsewhere in the genome (*37*).

#### Serogroups H and Z

The biochemical structures of serogroups H and Z contain monosaccharide glycerol-3-phosphate repeat units and share similarities with teichoic acid polymers. Region A varied from 6,182 bp in serogroup H to 7,198 bp in serogroup Z, with the latter containing the insertion element *IS1301*. Four genes were present in both serogroups (Figure and Technical Appendix Figure 3, panel A). BLASTp searches of the deduced amino acid sequences of the first 2 genes, termed *cshA/cszA* and *cshB/cszB*, shared 91% sequence identity with each other and also shared 76% and 63% sequence identity with the capsule biosynthesis genes *cps2B* and *cps2C* belonging to *Actinobacillus pleuropneumoniae* serovars 2, 3, 6, 7, 9, 11, and 13 (*38*). These genes are predicted to encode a glycerol-3-phosphate cytidylyltransferase, and a hypothetical protein containing a LicD domain for a role in phosphorylcholine incorporation into teichoic acid polymers has been suggested (*38*). The third genes in region A, termed *cshC* and *cszC*, each shared 65% sequence identity with a putative teichoic synthase genes (*cps2D* and *cps9D*, respectively) belonging to *A. pleuropneumoniae* serovars 2, 7, 9, and 13. Last, *cshD* and *cszD* were 75% homologous to *A. pleuropneumoniae* capsule synthesis genes (*cps7E*).

#### Serogroups L and X

Serogroup L capsule polysaccharides contain 2-acetamido-2-deoxy-D-glucosyl residues and phosphate groups. Region A contained 3 genes, *cslA*–*C* (formerly *lcbA*–*C*) and was 4,438 bp long. BLASTp searches of the deduced amino acid sequence from *cslA* predicted a capsule phosphotransferase sharing 73% sequence identity with LcbA proteins from *N. mucosa* C102 (GenBank accession no. ACRG00000000) and *N. subflava* NJ9703 (GenBank accession no. ACEO00000000), whereas *cslC* encoded an acetyltransferase protein. A gene similar to *cslA* was identified between regions D′ and B among serogroups I and K and a serogroup W isolate belonging to clonal complex sequence type 22 (Figure); BLASTp searches of the deduced amino acid sequence revealed that these genes shared 97% sequence identity. The longest gene, *cslB,* was 2,633 bp and putatively encoded a capsule polymerase (Figure and Technical Appendix Figure 2).

The serogroup X capsular polysaccharide is composed of (α-1→4)-linked *N-*acetylglucosamine 1-phosphate (*15*). In agreement with findings in a previous study, we found that region A in the serogroup X isolate contained 3 genes, *csxA*–*C* (*xcbA–C*) and was 4,467 bp long, including the insertion sequence, *IS1016,* located upstream of *csxA* (Figure and Technical Appendix Figure 2) (*19*). The deduced amino acid sequence from *csxA* shared 40% sequence identity with the previously mentioned LcbA protein belonging to *N. mucosa* C102, indicating that *csxA* encoded a putative capsule phosphotransferase; however, the remaining 2 serogroup X capsule biosynthesis genes did not share substantial sequence identities with any known protein.

#### Serogroups I and K

Different structural compositions have been described for these serogroups, with serogroup I consisting of *O-*acetylated alternating *N-*acetyl-guluronic acid and *N-*acetylmannosaminuronic acid units and serogroup K composed of *O-*acetylated disaccharide repeat units containing *N-*acetylmannosaminuronic acid (*10*,*11*). Both serogroups had almost identical capsule biosynthesis genes, *csiA*–*E* or *cskA*–*E*; region A was ≈9,026 bp long (Figure and Technical Appendix Figure 3, panel B). MLST analysis showed that serogroup I and K isolates investigated in this study did not possess the same sequence types or PorA and FetA variable regions (Table 1). Antiserum is not commercially available to verify these serogroups; however, the immunochemical difference between serogroup I and K polysaccharides may reflect differences in the original methods used to purify them (*10*,*11*). In addition, 2 nonsynonymous substitutions were observed in the capsule biosynthesis genes *csiC/cskC* and *csiD/cskD*, for which the deduced amino acid sequence encoded putative glycosyltransferases belonging to families 1 and 2, respectively. The capsule polymerases belonging to serogroups W and Y (*csw* and *csy*) are closely related; a single amino acid substitution in the *N-*terminal glycosyltransferase domain of the capsule polymerase produces the glucose or galactose substrate specificity for the enzyme (*39*) (online Technical Appendix). It is therefore possible that the nonsynonymous changes observed in the glycosyltransferases, csiC/cskC and csiD/cskD, may produce the serogroup I and K capsules. Further investigation of these serogroups is required.

The derived amino acid sequences from *csiA*, *B*, and *E/cskA*, *B*, and *E* shared >70% sequence identity with capsule biosynthesis proteins belonging to *Mannheimia haemolytica* serotype A1. The capsule of *M. haemolytica* serotype A1 is composed of a disaccharide repeat of *N-*acetylmannosaminuronic acid linked with *N-*acetylmannosamine.

### Regions B and C

The genes *ctrE* and *ctrF* (formerly *lipA* and *lipB*) are required for surface expression of a properly anchored capsule polymer. The ABC (ATP binding cassette) transport system is characterized by the hydrophobic outer and inner membrane proteins CtrA and CtrB, respectively; the integral inner membrane–associated protein, CtrC; and the ATP binding protein, CtrD, and homologous genes are found among other group II capsule-expressing bacteria (*40*) (see Technical Appendix).

## Conclusions

We compared nucleotide sequence data from complete *cps* loci from all described *N. meningitidis* serogroups, revealing that the genetic organization is similar for all loci and that region A contains the capsule-specific biosynthesis genes. Distinct capsule operons corresponding to serogroups A, B, C, E, H, I, K, L, W, X, Y, and Z have been described herein, with the serogroup D capsule described as being an unencapsulated serogroup C variant. Nucleotide sequence data for each capsule locus have been deposited in BIGS_DB_, with genes from each region defined and organized into schemes (*26*). However, it became apparent that a consistent approach to the nomenclature of capsule genes was required. In 2012, the approach detailed in this paper was approved at the XVIIIth International Pathogenic Neisseria Conference in Würzburg, Germany.

Horizontal genetic transfer of *cps* genes in region A was evident in serogroups H, I, K, and Z isolates, indicating acquisition of genes from external sources, including the bacterial species *A. pleuropneumoniae* and *M. haemolytica*. Combined with the low GC content observed in regions A and C, these data are consistent with acquisition of capsular genetic material from other species. Serogroup determination has been unresolved in some isolates. These isolates may contain capsule genes that are not expressed and will not be detectable by using conventional seroagglutination techniques, or they may be serogroup E, H, I, K, L, X, or Z, which are not routinely searched for and for which commercial antiserum is not available. This study provides additional tools to detect all capsule loci and may ultimately permit determination of the distribution of all serogroups among *N. meningitidis* populations and detection of *cnl* isolates.

Technical Appendix [Fig F1]Artemis comparison tool analyses of *Neisseria meningitidis*.
