# Supplementary material for: Description and Nomenclature of Neisseria meningitidis Capsule Locus
Source: Emerg Infect Dis. 2013 Apr;19(4):566–73. doi: 10.3201/eid1904.111799 (PMC3647402; doi:10.3201/eid1904.111799)
Supplement: Technical Appendix — Artemis comparison tool analyses of Neisseria meningitidis. [file 11-1799-Techapp-s1.pdf]

Article DOI: http://dx.doi.org/10.3201/eid1904.111799

Description and Nomenclature of *Neisseria meningitidis* Capsule Locus

Technical Appendix

A

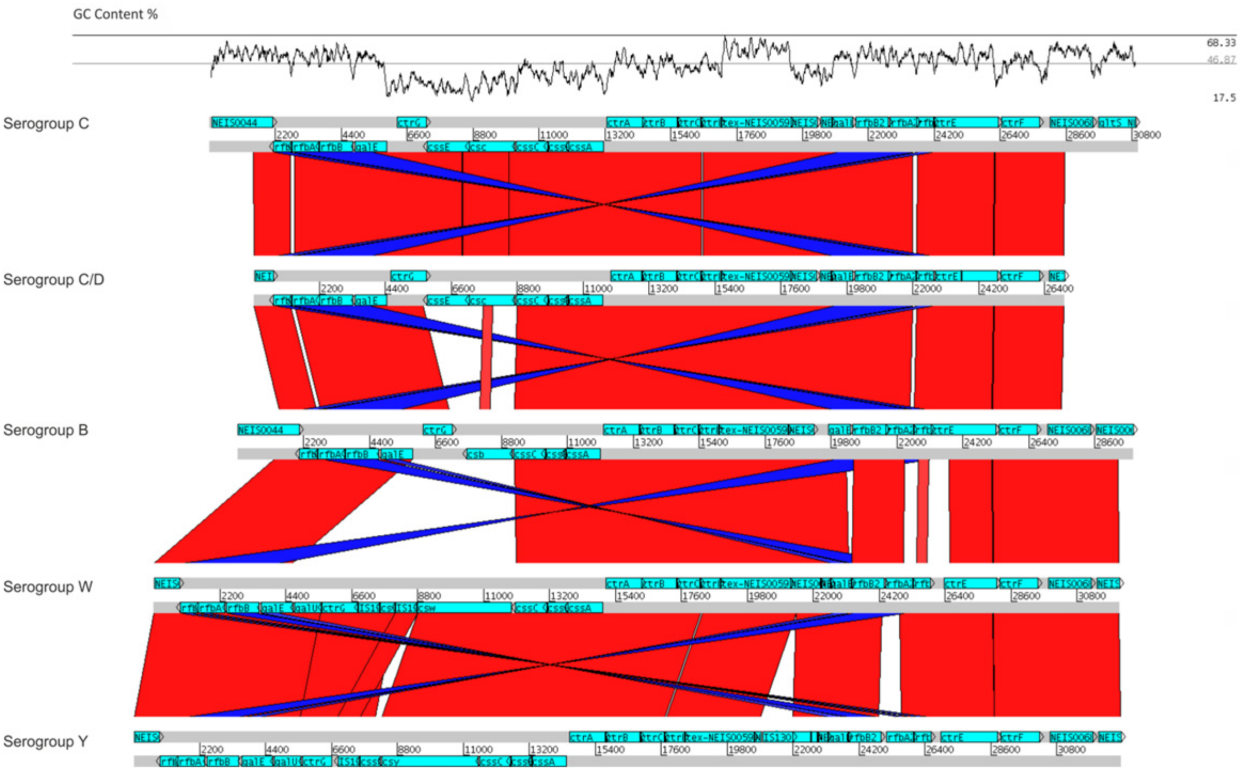

B

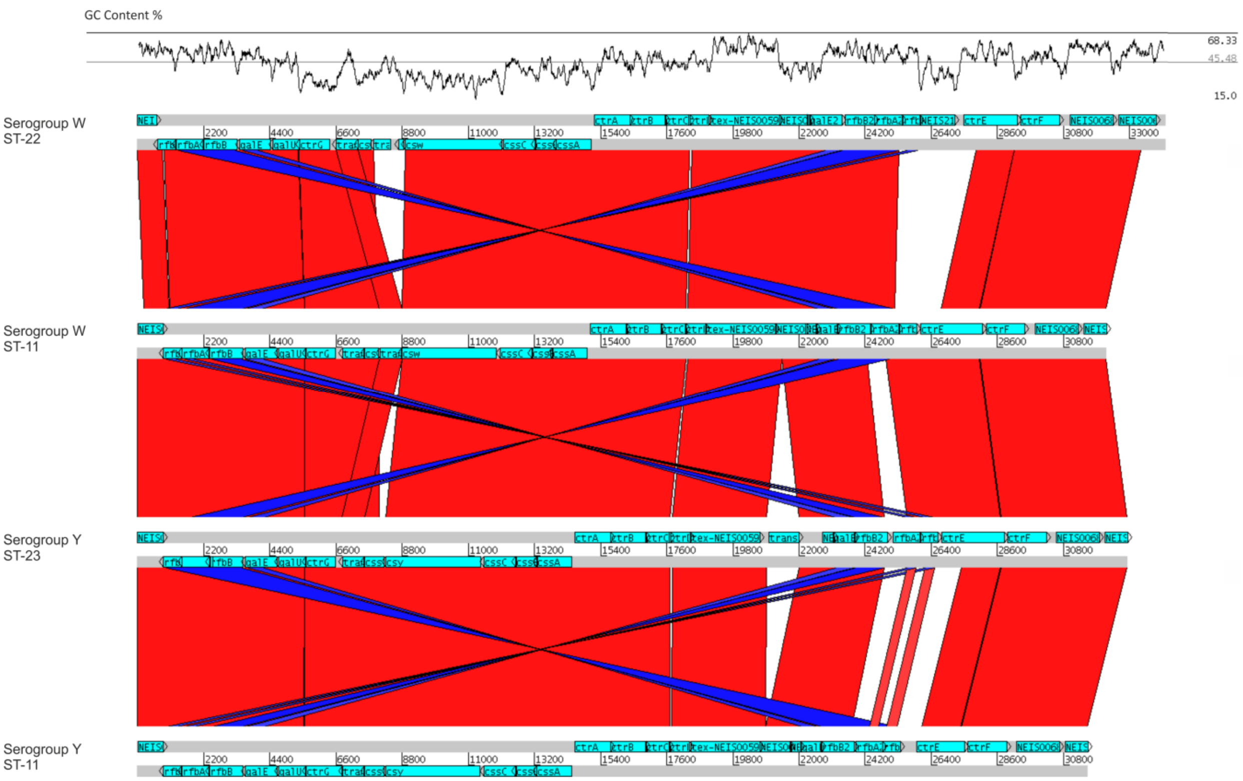

Figure 1. Panel A depicts a comparison of *Neisseria meningitidis* capsule loci from serogroups C, C (previously defined as serogroup D), B, W, and Y. Panel B displays a comparison of serogroups W and Y. The red and blue bands represent forward and reverse matches, respectively, with the intensity of the color proportional to the percentage sequence identity such that red indicates a good match (typically ranging from 90% to 100% sequence identity) and white indicates a low-scoring match. The average G+C content (%) across the bases is displayed at the top. Analyses were done by using the Artemis comparison tool (*27*).

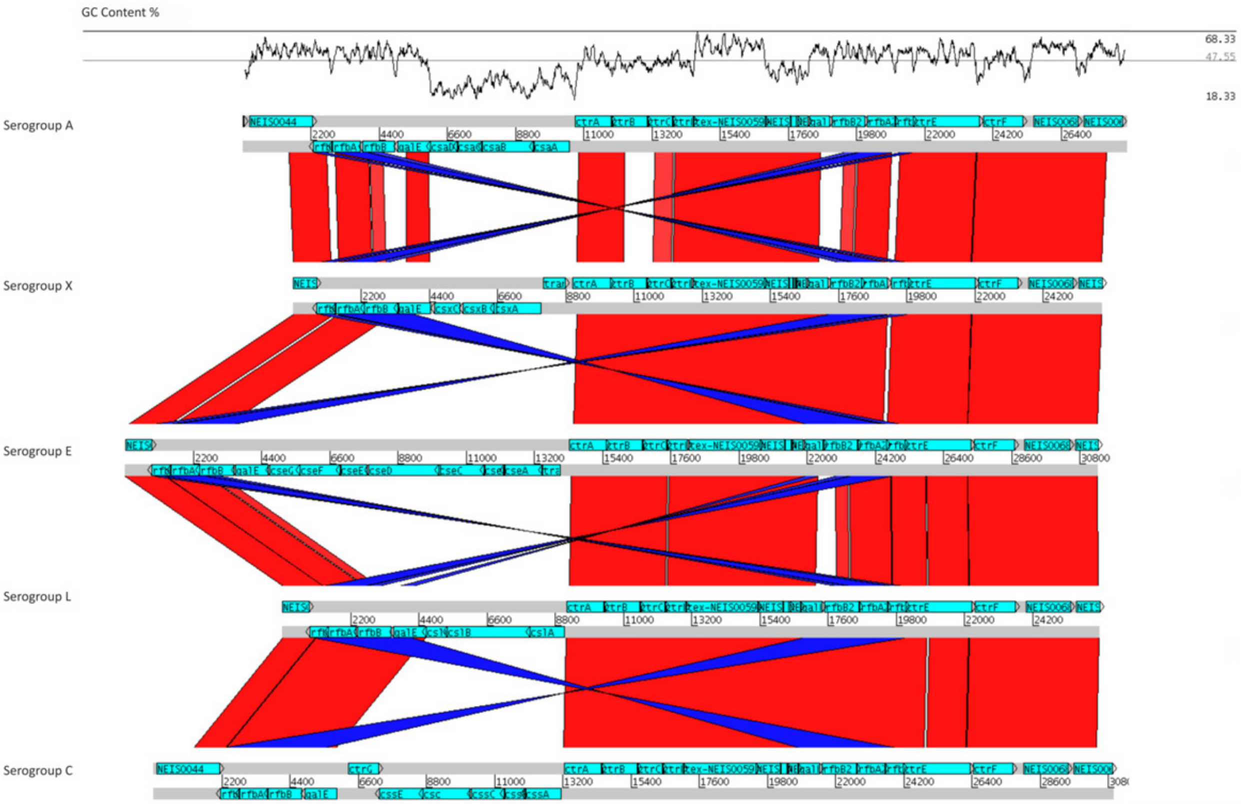

Figure 2. The figure compares *Neisseria meningitidis* serogroups A, X, E, L, and C. The red and blue bands represent forward and reverse matches respectively with the intensity of the color proportional to the percentage sequence identity such that red indicates a good match (typically ranging from 90% to 100% sequence identity) and white indicates a low-scoring match. The average G+C content (%) across the bases is displayed at the top. Analyses were done by using the Artemis comparison tool (*27*).

A

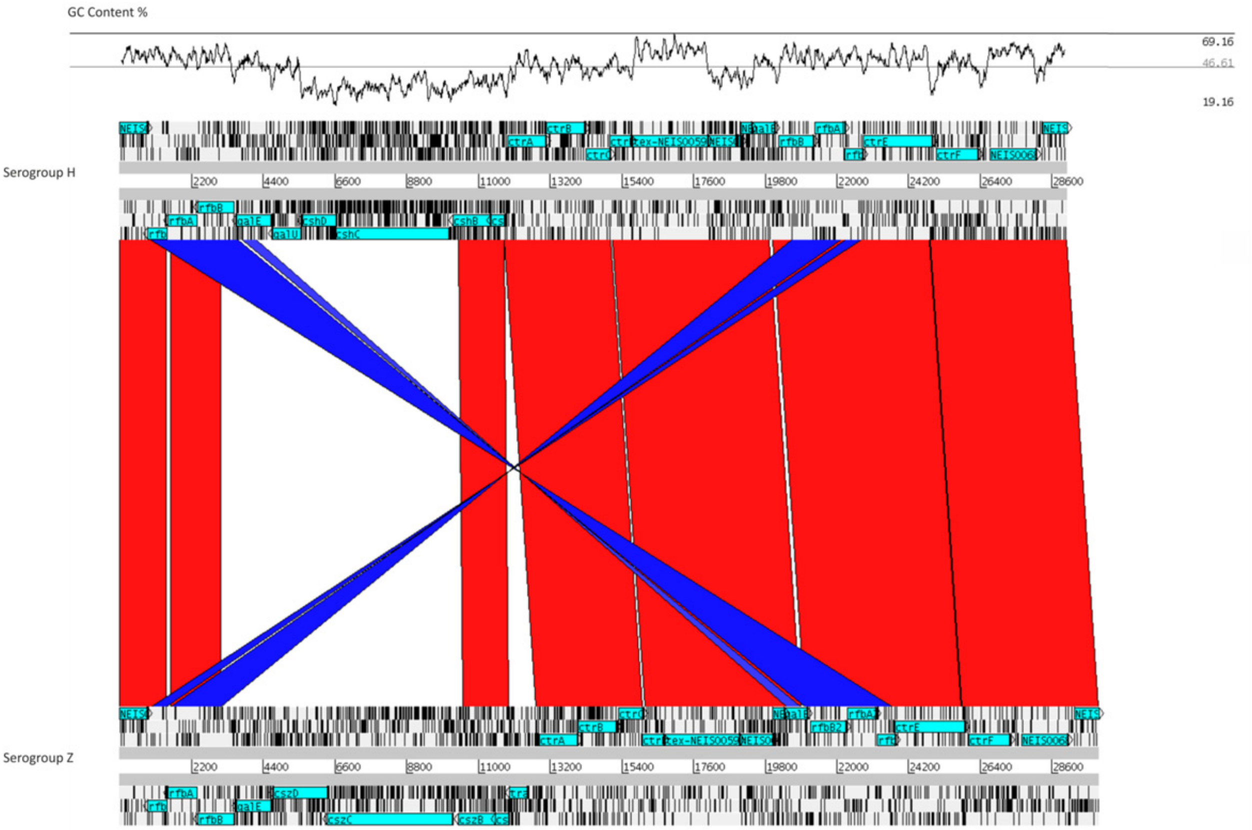
GC Content %
69.16
46.61
19.16
Serogroup H
2200
4400
6600
8800
11000
13200
15400
17600
19800
22000
24200
26400
28600
Serogroup Z
2200
4400
6600
8800
11000
13200
15400
17600
19800
22000
24200
26400
28600

B

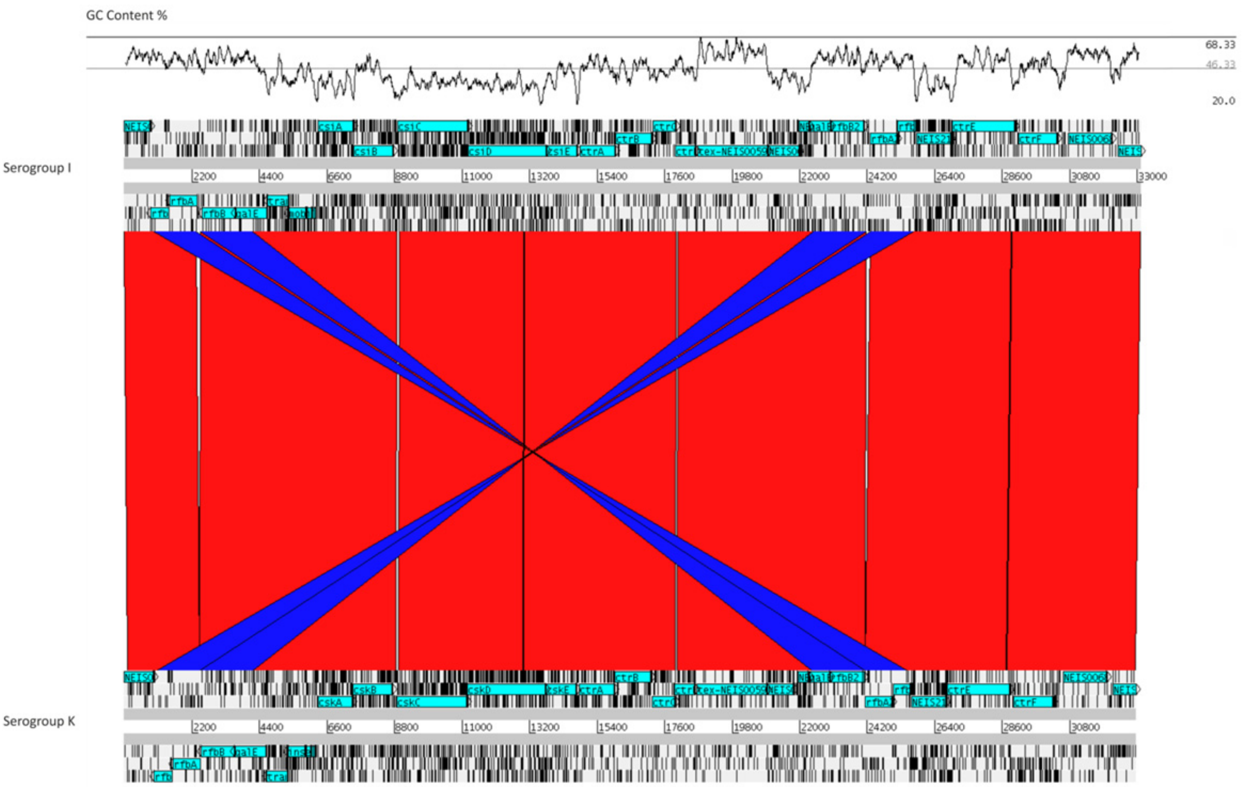

Figure 3. Panel A depicts *Neisseria meningitidis* serogroups H and Z. Panel B depicts serogroups I and K. The red and blue bands represent forward and reverse matches, respectively, with the intensity of the color proportional to the percentage sequence identity such that red indicates a good match (typically ranging from 90% to 100% sequence identity) and white indicates a low-scoring match. The average G+C content (%) across the bases is displayed at the top. Analyses were done by using the Artemis comparison tool (*27*).
